# Renal Thrombotic Microangiopathy Associated with the Use of Bortezomib in a Patient with Multiple Myeloma

**DOI:** 10.1155/2016/6020691

**Published:** 2016-05-16

**Authors:** Jan Van Keer, Michel Delforge, Daan Dierickx, Kathelijne Peerlinck, Evelyne Lerut, Ben Sprangers

**Affiliations:** ^1^Cardiology, University Hospitals Leuven, Herestraat 49, 3000 Leuven, Belgium; ^2^Hematology, University Hospitals Leuven, Herestraat 49, 3000 Leuven, Belgium; ^3^Vascular Medicine and Hemostasis, University Hospitals Leuven, Herestraat 49, 3000 Leuven, Belgium; ^4^Pathology, University Hospitals Leuven, Herestraat 49, 3000 Leuven, Belgium; ^5^Nephrology, University Hospitals Leuven, Herestraat 49, 3000 Leuven, Belgium

## Abstract

Bortezomib is a first-generation proteasome inhibitor used in the treatment of multiple myeloma (MM). A few reports have linked bortezomib exposure with the development of thrombotic microangiopathy (TMA). We describe a case of biopsy-proven renal thrombotic microangiopathy associated with the use of bortezomib in a 51-year-old man with IgG lambda MM. To our knowledge, this is the first biopsy-proven case. In addition, reexposure to bortezomib 18 months later was associated with recurrence of TMA. This supports a possible causal role of bortezomib. The exact mechanisms remain to be elucidated.

## 1. Introduction

Thrombotic microangiopathy (TMA) is a rare cause of renal disease in multiple myeloma (MM) patients. It is defined by microangiopathic hemolytic anemia, thrombocytopenia, and organ injury. Pathological features are arteriolar and capillary thrombosis with characteristic abnormalities in the endothelium and vessel wall [1]. TMA consists of a spectrum of multiple syndromes: thrombotic thrombocytopenic purpura (TTP), hemolytic-uremic syndrome (HUS), atypical HUS, and drug-induced TMA. TMA has been described in MM patients who received the proteasome inhibitor bortezomib [2–6]. In this report, we describe a case of renal TMA in a MM patient associated with exposure to bortezomib, with recurrence after reexposure.

## 2. Case Presentation

**Figure 1:**
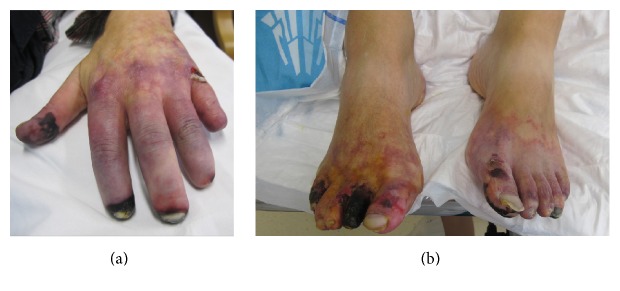
Acral ulcers. Painful, necrotising ulcers on hands (a) and feet (b). Note the amputation of distal phalanxes 4 and 5 of the left hand.

A 51 year-old Caucasian man was found to have acute kidney injury (AKI) three weeks after start of bortezomib- (1.3 g/m^2^) thalidomide- (100 mg) dexamethasone (40 mg) (VTD) therapy for newly diagnosed IgG kappa, Durie-Salmon stage IIIa, and ISS high risk multiple myeloma (MM). He had been diagnosed with monoclonal gammopathy of undetermined significance (MGUS) during the investigation of ulcerating acral skin lesions 9 years previously. M-protein level at diagnosis of MGUS had been 4.36 g/L. Workup for autoimmunity and cryoglobulinemia had been negative and biopsy of the skin lesions had shown nonspecific findings. The extent of the skin lesions seemed to correlate with the M-protein level. For this reason the patient had been treated with rituximab (7 doses of 375 mg/m^2^) and therapeutic plasma exchange (TPE). Other medical history included hypertension and hypothyroidism. His medications were amlodipine 5 mg, aspirin 80 mg, levothyroxine 75 *μ*g, and transdermal fentanyl 50 *μ*g/h. The patient had been smoking cigarettes for 30 years. The patient had received no other nephrotoxic medication and was well hydrated. Blood pressure was 150/83 mmHg. The patient's acral ulcers, which had been relatively well controlled with biweekly TPE for the last 9 years, had worsened, with development of a livedoid rash and painful edema of hands and feet and multiple necrotising ulcers (see Figure 1).

**Figure 2:**
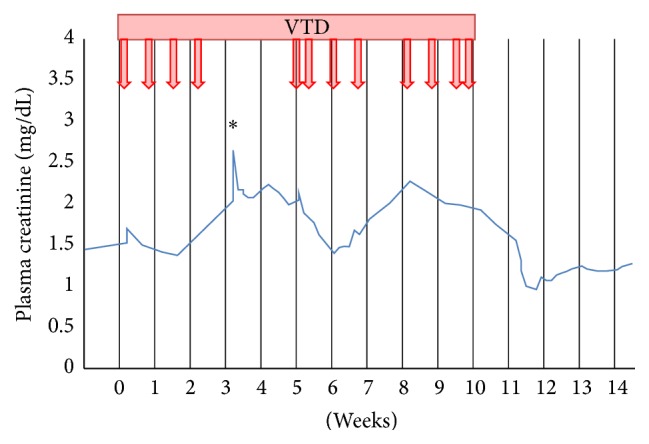
First episode of AKI. *x*-axis: time (weeks); 0 = start of first bortezomib administration; *y*-axis: plasma creatinine (mg/dL). VTD: bortezomib-thalidomide-dexamethasone; arrows: bortezomib administration; *∗*: time of renal biopsy.

**Figure 3:**
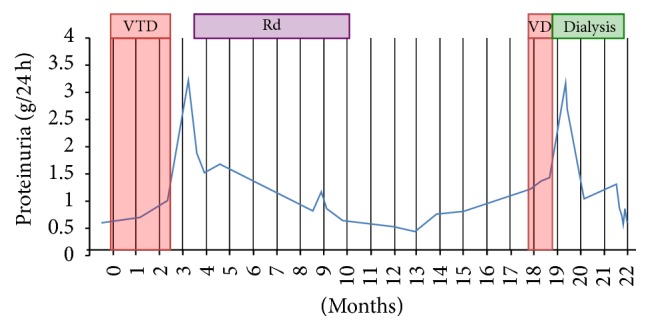
Evolution of proteinuria. *x*-axis: time (months); 0 = start of first bortezomib administration; *y*-axis: 24 h proteinuria (g/24 h). VTD: bortezomib-thalidomide-dexamethasone; Rd: lenalidomide-dexamethasone; VD: bortezomib-dexamethasone.

**Figure 4:**
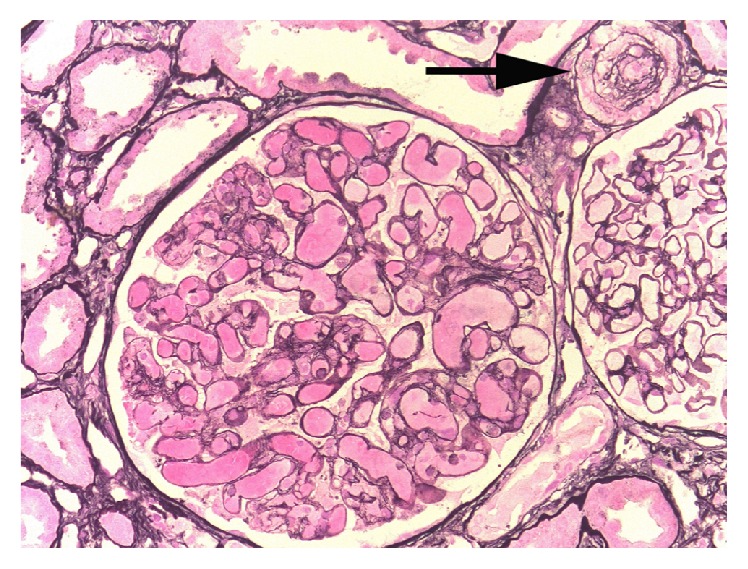
Renal biopsy. Renal biopsy showed 7 out of 36 obsolete glomeruli and 15% chronic tubulointerstitial damage. Note the capillary thrombus with intimal edema of the afferent arteriole (arrow).

Laboratory investigation showed a creatinine elevation from 1.3 mg/dL prior to start of VTD therapy to 2.7 mg/dL (see Table 1).  Figure 2 illustrates the relation between the onset of acute kidney injury and the administration of bortezomib. Proteinuria rose from 0.6 g/24 h before start of therapy to nephrotic range proteinuria, with a maximum of 3.2 g/24 h 3 months after start of VTD therapy (considerably later than the rise of creatinine had occurred; see Figure 3). There was dysmorphic hematuria (>2000 RBC/*μ*L). Platelet count was 119,000/*μ*L, Hb 7.5 g/dL with schistocyte excess on peripheral blood smear and haptoglobin < 0,1 g/L, indicative of microangiopathic hemolytic anemia. There was hypocomplementemia (C3 0.68 g/L, C3d 5.9%, and C4 0.14 g/L); cryoglobulins were absent; Coombs test, hepatitis B en C serology, ANF, and ANCA were negative. ADAMTS-13 activity was mildly reduced (34%). Genetic screening for complement mutations was negative. Renal sonogram revealed normal size kidneys with increased reflectivity of renal parenchyma. Punch biopsy of the livedoid skin rash showed nonspecific changes. Renal biopsy showed 7 out of 36 obsolete glomeruli and 15% chronic tubulointerstitial damage. One glomerulus had a capillary thrombus, with intimal edema of the afferent arteriole. These findings are indicative of a renal TMA lesion (see Figure 4). There were no arguments for paraprotein associated renal lesions, such as cast nephropathy, amyloidosis, light chain deposition disease, membranoproliferative glomerulonephritis, or cryoglobulinemia. Electron microscopy ruled out cryocrystalglobulinemia.

Based on the renal biopsy findings of TMA and the concomitant worsening acral ulcerations, TPE was intensified from biweekly to once every two days. Bortezomib was stopped after a total of 3 cycles, mainly because of the necrotising skin ulcers. Kidney function partially recovered to a new baseline creatinine of 2.1 mg/dL. Proteinuria diminished from 3.2 to 0.8 g/24 h over the following months. The patient was then started on lenalidomide- (15 mg) dexamethasone (40 mg). This therapy had to be stopped after 7 months due to pancytopenia, despite dose reduction. After 8 months of watchful waiting there was disease progression with both increase in serum paraprotein level and bone lesions on MRI. Based on the good initial hematological response to VTD, the patient was restarted on VD (bortezomib and dexamethasone). Note that, at that time, the possible link between TMA and bortezomib was not clear. Baseline creatinine before reexposure to bortezomib was 2.9 mg/dL. Other medications at that time included (total daily doses) nifedipine 120 mg, levothyroxine 75 *μ*g, omeprazole 40 mg, duloxetine 20 mg, amitriptyline 20 mg, calcium carbonate 1000 mg, cholecalciferol 880 E, and acetaminophen 4000 mg. Blood pressure was well controlled.

**Figure 5:**
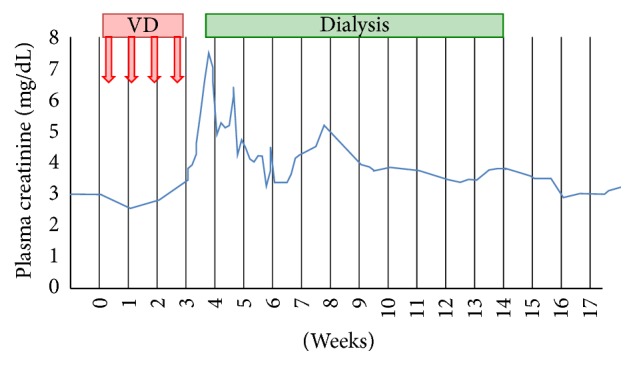
Second episode of AKI. *x*-axis: time (weeks); 0 = start of bortezomib rechallenge (18 months after first dose); *y*-axis: plasma creatinine (mg/dL). VD: bortezomib-dexamethasone; arrows: bortezomib administration.

Four weeks later, after 3 doses of bortezomib, the patient presented with a severe hypertension (188/102 mmHg), marked increase in serum creatinine (2.9 to 7.5 mg/dL; see Figure 5), macroscopic hematuria, proteinuria (1.2 to 2.7 g/24 h), microangiopathic hemolytic anemia (Hb of 7.9 g/dL), and severe thrombopenia (14,000/*μ*L). The patient was oliguric. He had excruciating pain in his extremities due to a flare up of the chronic ulcers. Because of fluid overload, acidemia, and hyperkalaemia, hemodialysis had to be started. TPE was again intensified to once every two days. After withdrawal of bortezomib, there was a gradual recovery. Proteinuria dropped again to 0.6 g/24 h.

Hemodialysis could be stopped after 2 months, with a new baseline creatinine of 3.2 mg/dL. Kidney function remained stable during 3 months of follow-up.

## 3. Discussion

We report a case of renal thrombotic microangiopathy (TMA) associated with the use of bortezomib in a multiple myeloma (MM) patient. Bortezomib is a first-generation proteasome inhibitor used in the treatment of MM. TMA has not been reported as a drug-related adverse event in CREST [7] and SUMMIT [8], the Phase 2 trials leading to bortezomib's approval. Ironically, bortezomib is even used successfully in the treatment of TTP (ADAMTS-13-deficiency-mediated TMA) [9], by depleting the causative antibodies. We identified five other cases of bortezomib-associated TMA in the literature, featuring various combinations microangiopathic hemolytic anemia and renal TMA. Morita et al. [2] and Moore and Romeril [3] described a picture of severe microangiopathic hemolytic anemia without significant renal insufficiency. Salmenniemi and Remes [4], Mehta et al. [5], and Chan et al. [6] described the same hematological picture, with renal insufficiency. In both cases a renal TMA lesion was suspected, but no kidney biopsy was performed. In Salmenniemi and Remes's case, a gingival biopsy was consistent with TMA. Ours is the first report of a documented renal TMA lesion after bortezomib exposure.

Carfilzomib, a second-generation proteasome inhibitor, has been linked to TMA in MM patients as well. Sullivan et al. [10] presented a patient with severe renal insufficiency (without biopsy) and thrombocytopenia. Hobeika et al. [11] described a case without significant renal insufficiency or thrombocytopenia, but with prominent hypertension and proteinuria, in which renal biopsy showed renal TMA and podocytopathy. Finally, Lodhi et al. [12] reported a case of MAHA with severe renal insufficiency in which renal biopsy showed features of TMA.

In all these reports the temporal relationship between exposure to bortezomib or carfilzomib and occurrence of TMA, and the improvement after discontinuation point towards proteasome inhibition as a possible cause of TMA. Our case is unique in that we documented recurrence of renal TMA after reexposure to bortezomib 18 months after the initial episode. This further supports a causal role for proteasome inhibition in the pathogenesis of renal TMA in multiple myeloma patients.

Drug-induced TMA can be caused by idiosyncratic (non-dose-related) immunologic reactions or by toxic (dose-related) effects [13]. The former type has been described after exposure to quinine [14], quetiapine [15], and gemcitabine [16] and might be attributed to endothelial damage by drug-dependent antibodies. The latter type has been described for many drugs, including antiplatelet, immunosuppressive, chemotherapeutic, and antiangiogenic agents [17]. Multiple reports of renal TMA have been described after exposure to bevacizumab. There is accumulating evidence that this could be a direct, on-target effect of the drug [18]. Eremina et al. [19] showed that survival of glomerular endothelial cells depends on VEGF from podocytes, by using conditional gene targeting: mice in which VEGF was selectively deleted from renal podocytes developed severe renal TMA. Although bortezomib has no direct effect on VEGF or its receptors, it has been shown that bortezomib triggers a dose-dependent inhibition of VEGF transcription via NF-*κ*B pathways [20–22]. This downregulation of VEGF could lead to damage to the glomerular microvasculature.

However, causality is difficult to establish. Hematopoietic stem cell transplantation, a known cause of TMA, could have played a role in 4 of the above-mentioned reports [2, 4, 11, 12]. Another possible culprit of TMA in MM patients is the paraprotein itself. TMA has been described in MM with an anti-ADAMTS-13 paraprotein [23]. In our case, each episode of AKI occurred after a rise in paraprotein levels: at diagnosis of MM and at disease progression. Moreover, renal function did not resolve completely after discontinuation of bortezomib, nor did the MAHA. Interestingly, the necrotising skin ulcers, which had started 9 years previously and were likely to be paraprotein-driven as they were rituximab and TPE responsive, flared up together with the two episodes of AKI. We therefore hypothesize that our patient had a latent vasculopathy, possibly paraprotein-induced, which predisposed him to development of TMA, possibly provoked by bortezomib-induced VEGF-deficiency.

Treatment of drug-induced TMA is limited to drug avoidance and supportive care [17]. Some authors propose TPE [10]. TPE is an established therapy for TTP, restoring ADAMTS-13 activity by eliminating the causative antibodies. As the pathophysiology of drug-induced TMA is different from TTP, TPE is less likely to be beneficial. We intensified the on-going TPE therapy in our patient because of the favourable effect on his skin lesions. Whether this had a positive effect on the TMA is uncertain.

## 4. Conclusion

We report a case of renal TMA in a MM patient who is treated with bortezomib. This is the sixth case of TMA associated with bortezomib exposure in the literature. To our knowledge, this is the first biopsy-proven case. In addition we show that reexposure to bortezomib caused recurrence of TMA 18 months later. The possible mechanisms remain to be elucidated.

## Figures and Tables

**Table 1:** Laboratory values.

	*t* = 0 (Start BTZ)	*t* = +3 w (Time of renal biopsy)	*t* = +4 m (1 m after stop of BTZ)	*t* = +18 m (Rechallenge BTZ)	*t* = +19 m (Start dialysis)	*t* = +22 m (After stop of dialysis)
Hb (g/dL)	9.2	7.5	9.2	9.8	7.9	8.7
WBC (×10^9^/L)	2.43	1.37	2.19	2.63	3.07	2.37
Platelets (×10^9^/L)	128	119	77	34	14	42
Schistocytes (/1000 RBC)	<3	4–7	<3	<5	40–50	<5
Haptoglobin (g/L)	—	<0.1	3.08	1.33	<0.1	0.9
LDH (U/L)	156	218	102	468	419	237
Creatinine (mg/dL)	1.35	2.65	2.04	3.00	7.49	2.89
Ureum (mg/dL)	39	70	33	80	147	97
Proteinuria (g/24 h)	1.2	0.6	1.52	1.37	2.69	0.6
Hematuria (RBC/*µ*L)	258	2079	661	—	Hematuria	Hematuria
Pyuria (WBC/*µ*L)	14	24	3	—	56	4
IgG (g/L)	88.2	—	24.3	16	10.7	—

BTZ: bortezomib; w: weeks; m: months; —: not available.
